# Corneal transplantation activity in Catalonia, Spain, from 2011 to 2018: Evolution of indications and surgical techniques

**DOI:** 10.1371/journal.pone.0249946

**Published:** 2021-04-08

**Authors:** Noelia Sabater-Cruz, Marc Figueras-Roca, Lydia Padró-Pitarch, Jaume Tort, Ricardo P. Casaroli-Marano

**Affiliations:** 1 *Institut Clinic d’Oftalmologia* (ICOF), Hospital Clinic de Barcelona, Barcelona, Spain; 2 Catalan Transplant Organization (*Organització Catalana de Trasplantaments—*OCATT), Barcelona, Spain; 3 Barcelona Tissue Bank (BTB), *Banc de Sang i Teixits* (BST), Barcelona, Spain; 4 Department of Surgery, School of Medicine and Health Sciences, University of Barcelona, Barcelona, Spain; University of Toronto, CANADA

## Abstract

**Purpose:**

To report corneal transplant activity carried out in Catalonia (Spain) and the evolving indications for keratoplasty over an 8-year period.

**Methods:**

Annual reports from the Catalan Transplant Organization, Spain, on corneal graft indications and techniques from 2011 to 2018 were reviewed.

**Results:**

A total of 9457 keratoplasties were performed in Catalonia, from January 2011 to December 2018. The most frequent indications were bullous keratopathy (BK; 20.5%), Fuchs endothelial dystrophy (FED; 17.9%), re-graft (13.7%), and keratoconus (11.3%). Penetrating keratoplasty (PKP) accounted for 63.4% of all performed keratoplasties. Since the introduction of eye bank precut tissue for Descemet stripping automated endothelial keratoplasty (DSAEK) in 2013 and for Descemet membrane endothelial keratoplasty (DMEK) in 2017 the number of endothelial keratoplasties has drastically increased. An increasing trend of posterior lamellar techniques over the total of keratoplasties was found (p<0.001). Endothelial keratoplasties for different endothelial diseases indications (BK, FED, and re-graft), also showed and increasing trend (p<0.001). DMEK is the technique with the highest increase (statistically significantly different from linearity) over other endothelial keratoplasties in FED (p<0.001) but not in BK (p = 0.67) or re-grafts (p = 0.067).

**Conclusion:**

Endothelial diseases represented the top indication for keratoplasty over the 8-year period. PKP is still the most used technique in Catalonia, but endothelial keratoplasties and especially DMEK showed a significant increasing trend over the last years. This is congruent with the main rationale nowadays for keratoplasties: to customize and transplant as less tissue as possible. Therefore, the availability of precut tissue could have definitely enforced such approach.

## Introduction

Corneal grafts are one of the most frequent transplanted tissues in the world [1]. In addition, keratoplasty techniques, and therefore indications, have majorly changed these last years, thanks to the upraise of selective corneal surgeries that have vastly improved visual recovery results. However, epidemiology on such surgeries is not always easy and mostly depends on regional eye bank report services. Moreover, several studies suggest most surgeons tend to customize indications and even perform more posterior lamellar techniques when eye bank precut tissue is available [2,3].

In 1989 the Spanish National Transplant Organization (*Organización Nacional de Trasplantes*–ONT) started its activity coordinating organ and tissue donations in Spain. In 1994, the autonomous region of Catalonia, started its own organization (*Organització Catalana de Trasplantaments*–OCATT), and joined efforts with ONT [4]. However, keratoplasty indications, techniques and trends have not been studied in depth since the introduction of precut corneal tissue supply by the main regional eye bank in such a population with a high volume of transplantations, 1000 keratoplasties/year on average, representing 25% to 30% of total keratoplasties in Spain. Over the last few years, the number of keratoplasties has increased in Spain (over 4000 keratoplasties/year since 2016, and even more in Catalonia [5].

In Catalonia, retrieval of ocular tissue is mainly performed by transplant coordinator teams and centralized by an eye bank (Barcelona Tissue Bank, BTB) [6]. Then, the ocular tissue is processed and distributed. The OCATT collects information on tissue viability and distribution from the different transplant centres, and surgical and clinic details and keratoplasty technique indications from standardized questionnaires sent to surgeons. Moreover, OCATT has a biovigilance system to detect adverse events and adverse reactions.

Being a centralized eye bank in Catalonia, BTB supplies corneal grafts an offers precut tissue for posterior lamellar techniques. In 2013, it started to supply precut tissue for Descemet Stripping Automated Endothelial Keratoplasty (DSAEK), and from 2017, precut tissue for Descemet Membrane Endothelial Keratoplasty (DMEK) was distributed [6]. Despite few surgeons started to perform endothelial keratoplasties (EK) before 2013, from that date, more and more surgeons dared to switch to posterior lamellar techniques due to the availability of precut tissue [3].

This study investigates the evolving trends of corneal transplantation from 2011 to 2018 in Catalonia centres, and its relationship with precut tissue supply.

## Materials and methods

This study is a retrospective review of corneal tissue activity records of the OCATT in Catalonia, between January of 2011 and December of 2018. The included data corresponded to corneal tissue retrieved and implanted in Catalonia. ONT website was consulted to obtain data regarding the Spanish registries [5]. Institutional Ethics Committee Board approval was obtained for donor data revision (approval number HCB/2015/0879, Hospital Clinic de Barcelona, amended on 14th November of 2018). Research methods and analysis plan adhered to the Tenets of the Declaration of Helsinki. Data related to ocular tissue, its traceability, and potential adverse events were treated in accordance with the appropriate European Union directives (2004/23/EC, 2006/17/EC, and 2006/86/EC). Patient data were encoded for management in accordance with the Spanish legislation on personal data protection (RD05/2018).

Reported data outcomes included, as per every year, number of cases in all surgical techniques as well as number of cases in each approach by their main clinical indication for keratoplasty. Descriptive results are presented as absolute frequencies and percentages for categorical variables. Linear trends (Mantel-Haenszel statistic) and deviation from linearity were obtained. All tests were performed with a two-sided type I error of 5% with the statistical package STATA v.15.1 (StataCorp, College Station, Texas, USA).

## Results

**Fig 1:**
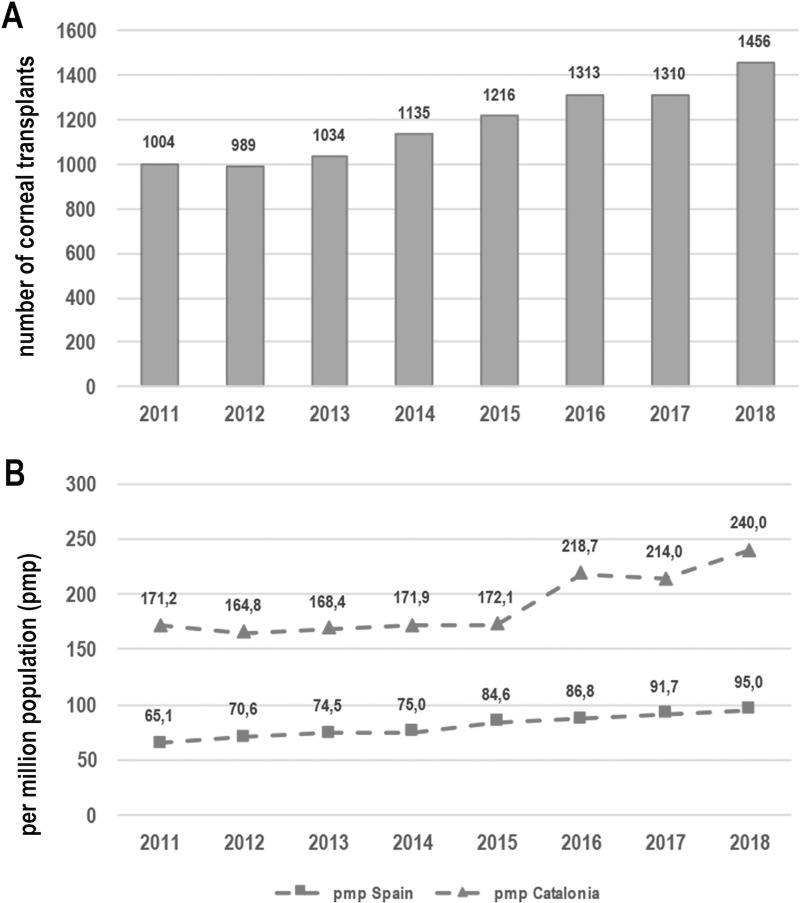
Number of keratoplasties per year carried out in Catalonia (1A) and corneal transplants per million inhabitants in Catalonia and Spain (1B).

Between 2011 and 2018, 9457 keratoplasties were performed in Catalonia, that is 1182 keratoplasties/year on average (ranging 989–1456 per year) (Fig 1A). To report how prevalent or frequent are transplants among regions or countries, the unit transplants per million population (pmp) is commonly used. In this regard, 190.2 corneal transplants per million population and year were performed on average in Catalonia (Fig 1A), while this figure was 80.4 pmp/year on average in Spain (Fig 1B).

Together with the corneal tissue sample, a follow-up form is sent to the surgeon, asking for adverse events, surgical technique, and surgery indication, among others. Despite the high recommendation to send it back, the response rate per year was heterogeneous, ranging from 59.4% to 96%. Information about surgical indication and keratoplasty technique was obtained from 7660 out of 9457 procedures (80.9%). The indications for corneal transplantation are listed in Table 1. The most frequent indications were bullous keratopathy (BK; 1574; 20.5%), Fuchs endothelial dystrophy (FED) and other endothelial dystrophies (1373; 17.9%), re-graft due to endothelial failure (1051; 13.7%), and keratoconus (865; 11.3%).

**Table 1:** Indications reported for corneal transplantation from January 2011 to December 2018 in Catalonia (Spain).

Diagnosis	Number of transplants (%)
Bullous keratopathy secondary to surgery (any)	1574 (20.5)
Fuchs endothelial dystrophy	1373 (17.9)
Re-graft due to endothelial failure	1051 (13.7)
Keratoconus/ectasia (other than post-refractive)	865 (11.3)
Infection (viral, fungal and bacterial)	604 (7.9)
Corneal degeneration	563 (7.3)
Chemical injury/trauma	381 (5.0)
Congenital opacity	225 (2.9)
Ulcerative keratitis (non-infectious)	221 (2.9)
Stromal corneal dystrophy	213 (2.8)
Refractive surgical complication	50 (0.7)
Other*	540 (7.0)
Total of transplants with diagnosis informed	7660 (100)
Total of transplants	9457

*Other included: Rejection (134; 1.75%), regraft for a reason other than endothelial failure (78; 1%), oedema of unknown origin (67; 0.9%), irregular astigmatism (22; 0.3%), opacification (14; 0.18%), perforation (9; 0.12%), toxic epidermal necrolysis (7; 0.09%), descemetocele (5; 0.06%), pemphigoid (4; 0.05%), Peters anomaly (2; 0.03%), congenital glaucoma (1; 0.01%), bacterial keratitis and perforation (1; 0.01%), epithelial ingrowth (1; 0.01%), hematocornea (1; 0.01%), unknown (194; 2.5%).

**Fig 2:**
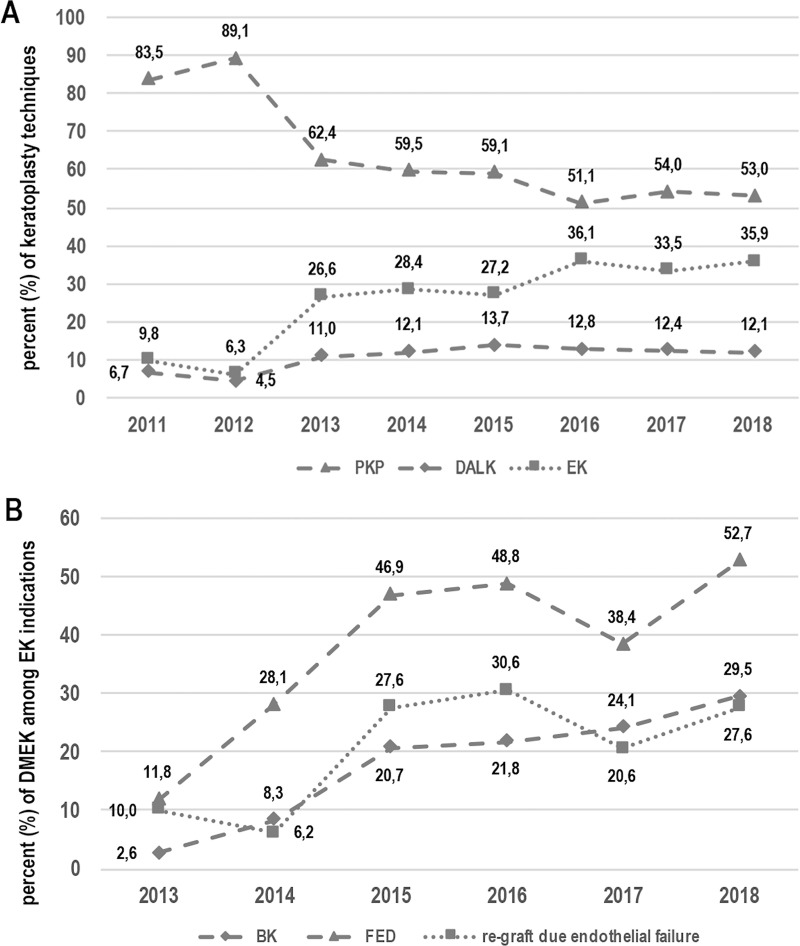
Corneal transplantation techniques: Evolving trends in the period of 2001–2018 (2A). Trends of DMEK in endothelial keratoplasties from 2013 to 2018 for different indications–BK, FED, and re-grafting due to endothelial failure (2B). DMEK: Descemet Membrane Endothelial Keratoplasty. PKP: Penetrating Keratoplasty, DALK: Deep Anterior Lamellar Keratoplasty, EK: Endothelial Keratoplasty. BK bullous keratopathy. FED: Fuchs Endothelial Dystrophy.

Corneal transplantation techniques performed from 2011 to 2018 were: penetrating keratoplasty (PKP; 4848; 63.4%), deep anterior lamellar keratoplasty (DALK; 827; 10.7%), and endothelial keratoplasty (1985; 25.9%). During the years 2011 and 2012, due to the low number of posterior lamellar keratoplasties performed, no distinction between DMEK and DSAEK was formally made. Evolving trends in keratoplasty techniques over years are shown in Fig 2A.

The evolving trend of lamellar techniques was statistically analysed and resumed in Table 2. It showed statistically significant increasing trends of endothelial and anterior lamellar keratoplasties, which differ from linearity in almost all reported associations but for DMEK performed on BK, given the low number of cases. In addition, although DMEK showed an increasing trend in re-grafts, it did not reach statistical significance (p = 0.067) (Fig 2B).

**Table 2:** Increasing trends of different techniques globally and for different pathologies (over total of keratoplasties with available information).

Technique analysed	n/total analysed	% in 2011 (2013 for DMEK, in italics)	% in 2018	Increasing trend	Different from linearity
**Overall keratoplasties (2011–2018)**					
EK over total of keratoplasties	1985/7660	9.8	36.0	p<0.001	p<0.001
EK in endothelial diseases*	1825/4003	2.0	54.9	p<0.001	p<0.001
EK in BK	588/1576	19.0	29.9	p<0.001	p<0.001
EK in FED	999/1377	43.2	92.0	p<0.001	p<0.001
EK in re-grafts	228/1030	4.1	36.7	p<0.001	p<0.001
DALK over total of keratoplasties	827/7660	6.8	12.2	p<0.001	p<0.001
DALK in ectasia and KC	359/865	23.6	59.4	p<0.001	p = 0.0129
**Overall EK (2013–2018)**					
DMEK over EK	563/1835	*7*.*4*	43.8	p<0.001	p = 0.02
DMEK over EK in BK	96/513	*2*.*6*	29.5	p<0.001	p = 0.67
DMEK over EK in FED	376/938	*11*.*8*	52.7	p<0.001	p<0.001
DMEK over EK in re-grafts	50/214	*10*.*0*	27.6	p = 0.067	NA

EK: Endothelial keratoplasty; DMEK: Descemet membrane endothelial keratoplasty; BK: Bullous keratopathy; FED: Fuchs endothelial dystrophy; NA: Non-applicable; KC: Keratoconus.

*Endothelial diseases: BK, FED, and re-graft due to endothelial failure.

## Discussion

Catalonia is an autonomous region of Spain with 32108.2 km^2^ and a population of 7.5 million people at end of 2018 [7]. The number of corneal transplants performed in Catalonia from 2011 to 2018 has been 9457 (average of 1050.7 keratoplasties/year), corresponding to 190.2 corneal transplants per million population and year performed on average, while this figure is 80.4 on average in Spain. In other words, 32.7% of all keratoplasties performed in Spain were performed in Catalonia [5]. Currently, the BTB is the only bank in Spain that processes corneas for precut tissue delivery [8].

As observed, the most usual indication for corneal transplantation during the period of study was BK followed by FED. The most prevalent technique was PKP, which accounted for 63.4% of all performed keratoplasties. A statistically significantly increasing trend of posterior lamellar techniques over the total of keratoplasties was found. In addition, DMEK was indeed the technique with the highest increase over other endothelial keratoplasties in FED but not in BK or re-grafts due to endothelial failure. That increase could be associated to the start of precut tissue supply for EK by the regional corneal tissue bank. Therefore, a predominance of the lamellar techniques is expected to be found in the next years. Interestingly, such a shift has already happened in other countries several years earlier than in the studied area: PK replaced by lamellar keratoplasties was first reported in 2015 in the United States [9] and 2017 in Germany [10] and a significant shifting trend was stated in 2014 in Canada [11,12] and 2015 in Italy; [13] while in other areas this shift had not been detected so far [14].

Indications for corneal transplantation are still different from one region to another, despite overall trends are indeed changing and converging: BK and FED have been seen to be most common indications for transplant in developed countries whereas infective keratitis and scars are more common in developing countries [11,15–17]. However, BK may represented the second indication—after FED [9,11,12,18] or keratoconus [19,20]—and even the third one [10]. Intrinsic methodological issues such as questionnaire codification—for example FED with oedematous cornea could be eventually codified as BK—could explain that finding over the sole fact of a lower prevalence of FED in that geographical area per se.

Keratoplasty nowadays sticks to the rationale of transplanting as less tissue as possible in order to avoid allograft rejection, reduce postoperative complications and increase graft survival rates. Among endothelial techniques, DSAEK receptors often have a hyperopic shift and, in some cases, suboptimal visual recovery [21–23] while DMEK is a more anatomically accurate procedure that just replaces Descemet membrane and endothelium, potentially leading to a faster and better visual recovery with minimal refractive change [24–27]. Therefore, DMEK could be considered as the intended common-practice technique for corneal endothelial disease but, however, its widespread adoption is limited due to the overall challenging surgical technique in addition to the relative difficulty of donor tissue preparation [2,25].

The availability of precut tissue for DSAEK started in Catalonia in 2013, which representing a breakthrough in EK surgical indication [6]. This shift was even more important after the introduction of precut tissue for DMEK in 2017 [6]. Some studies [3] have already highlighted an increased trend to perform EK after precut tissue introduction. It is commonly thought that surgeons are more prone to perform posterior lamellar techniques when the risk to damage corneal tissue—due to manual preparation—is very low [28,29]. Previous reports have shown no differences in best corrected visual acuity, central corneal pachymetry or complications—dislocations, primary graft failure—found between corneas prepared by the surgeon in the operating room and precut tissue for DSAEK [29–31] or even DMEK [32]. Other advantages of precut tissue are the lower rate of microbiological culture positivity and lower risk of receptor infection [33,34]. Despite that, some areas still favour DMEK technique regarding indications over DSAEK even without precut tissue [10]. Taking into consideration all these facts and figures we are prone to presume than in the next few years DMEK trend in Catalonia will continue its increase thanks to precut tissue, among other reasons. Despite that, the increasing trend in lamellar techniques used in Catalonia has turn up late compared to other countries: for example, in 2011 in Catalonia only 19% of BK were treated with EK whereas in Columbia (Canada) this proportion corresponded to 57.7%; [11] in the United States of America, about 50% of all corneal transplants are indeed EK according to the Eye Bank Association of America [9,35] while in this study that figure only accounted for 9.7%. Moreover, in Italy, an increasing trend of posterior lamellar techniques was found between 2002 and 2008 whereas in our area it happened after 2013 [13]. Other authors have investigated the keratoplasty activity in different centers in Spain [36,37]. As in their conclusions, we found than PKP was still the most prevalent technique used globally during the period of study despite the advantages described in literature of EK over PKP [21,23,25].

Even though being based on solid and official data on tissue transplantation, this study has still some limitations to disclose. First, as it is based on the OCATT’s annual reports, data is kept subject to surgeons’ responses accuracy. In other studies, preoperative diagnosis reported by surgeons ranged from 50% to 97% [9,38]. Surgeons must be aware of the importance of returning follow-up forms in order to increase exactness of reports. In addition, starting of any supply procedure, such as corneal precut tissue, can present with logistic problems such as bureaucracy issues or temporal shortage; therefore, data on usage of such supply could have been underestimated.

Generally, EK have shown several determinant advantages over PKP such as better visual acuity outcomes, less rejection rate and better postoperative recovery, despite being technically more difficult and having a higher learning curve [15,39,40]. These proven benefits in many prevalent indications and the extended use of eye bank precut tissue are thought to be the main causes of the increasing trend of both EK as a whole and DMEK in particular in our area. Given these characteristics in the next few years we could expect EK to drastically outperform PKP in endothelial corneal disease. On the other hand, and for similar reasons, we could also presume an overall EK major shift towards DMEK accordingly. In-depth epidemiological studies as the presented one would be mandatory in the years to come to follow-up and evaluate tissue transplantation trends.

## References

[1] Gadhvi KA, Coco G, Pagano L, Kaye SB, Ferrari S, Levis HJ, et al. Eye Banking: One Cornea for Multiple Recipients. Cornea. 2020;39:1599–1603. 10.1097/ICO.0000000000002476 32947412

[2] Terry MA. Endothelial Keratoplasty: Why Aren’t We All Doing Descemet Membrane Endothelial Keratoplasty? Cornea. 2012;31:469–471. 10.1097/ICO.0b013e31823f8ee2 22367047

[3] Palma-Carvajal F, Morales P, Salazar-Villegas A, Figueroa-Vercellino JP, Spencer F, Peraza-Nieves J, et al. Trends in corneal transplantation in a single center in Barcelona, Spain. Transitioning to DMEK. J Fr Ophtalmol. 2020;43:1–6. 10.1016/j.jfo.2019.06.026 31831273

[4] Organització Catalana de Trasplantaments. History of transplants in Catalonia [Internet]. Available from: http://trasplantaments.gencat.cat/ca/ocatt/historia/.

[5] Organización Nacional de Trasplantes. Specialized information and reports about donation and transplantation [downloadable pdf]. Available from: http://www.ont.es/infesp/Memorias.

[6] Sabater-Cruz N, Otero N, Dotti-Boada M, Ríos J, Gris O, Güell JL, et al. Eye bank and theatre factors for positive microbiological culture of corneoscleral rim and cornea storage medium in the real-world. Eye. 2021. 10.1038/s41433-020-01342-8 33469128 PMC8526809

[7] Gencat: Generalitat de Catalunya. Informació, Tràmits i serveis de la Generalitat de Catalunya. Topics regarding Catalonia [accessed on October 26, 2019]. Available from: https://web.gencat.cat/ca/temes/catalunya/.

[8] Banc de Sang i Teixits. Professionals area [Internet]. Available from: https://www.bancsang.net/professionals/teixits/58/lamel·la.

[9] Park CY, Lee JK, Gore PK, Lim C, Chuck RS. Keratoplasty in the United States A 10-Year Review from 2005 through 2014. Ophthalmology. 2015;122:2432–2442. 10.1016/j.ophtha.2015.08.017 26386848

[10] Röck T, Landenberger J, Bramkamp M, Bartz-schmidt KU, Röck D. The Evolution of Corneal Transplantation. Ann Transplant. 2017;22:749–754. 10.12659/aot.905498 29242495 PMC6248302

[11] Tan JCH, Holland SP, Dubord PJ, Moloney G, Mccarthy M, Yeung SN. Evolving Indications for and Trends in Keratoplasty in British Columbia, Canada, from 2002 to 2011: A 10-year Review. Cornea. 2014;33:252–256. 10.1097/ICO.0000000000000066 24457452

[12] Zhang AQ, Rubenstein D, Price AJ, Elie C, Levitt M, Sharpen L, et al. Evolving surgical techniques of and indications for corneal transplantation in Ontario: 2000–2012. Can J Ophthalmol. 2013;48:153–159. 10.1016/j.jcjo.2012.12.008 23769775

[13] Frigo AC, Fasolo A, Capuzzo C, Fornea M, Bellucci R, Busin M, et al. Corneal Transplantation Activity Over 7 Years: Changing Trends for Indications, Patient Demographics and Surgical Techniques From the Corneal Transplant Epidemiological Study (CORTES). Transplant Proc. 2015;47:528–535. 10.1016/j.transproceed.2014.10.040 25769602

[14] Wang TW, Chi YC, Hsu PS, Kuo NW, Chen JL. Changing Indications for Corneal Transplantations in Southern Taiwan From 2008 to 2018. Eye Contact Lens. 2020;46:301–305. 10.1097/ICL.0000000000000638 31313699

[15] Singh R, Gupta N, Vanathi M, Tandon R. Corneal transplantation in the mordern era. Indian J Med Res. 2019;150:7–22. 10.4103/ijmr.IJMR_141_19 31571625 PMC6798607

[16] Gupta N, Vashist P, Tandon R, Gupta SK, Dwivedi S, Mani K. Prevalence of corneal diseases in the rural Indian population: the Corneal Opacity Rural Epidemiological (CORE) study. Br J Opthtalmology. 2015;99:147–152. 10.1136/bjophthalmol-2014-305945 25395684

[17] Chen W, Hu F, Wang I. Changing Indications for Penetrating Keratoplasty in Taiwan from 1987 to 1999. Cornea. 2001;20:141–144. 10.1097/00003226-200103000-00004 11248815

[18] Le R, Yucel N, Khattak S, Yucel YH, Prud’homme GJ, Gupta N. Current indications and surgical approaches to corneal transplants at the University of Toronto: A clinical-pathological study. Can J Ophthalmol. 2017;52:74–79. 10.1016/j.jcjo.2016.07.005 28237153

[19] Fasolo A, Frigo AC, Böhm E, Genisi C, Rama P, Spadea L, et al. The CORTES Study: Corneal Transplant Indications and Graft Survival in an Italian Cohort of Patients. Cornea. 2006;25:507–515. 10.1097/01.ico.0000214211.60317.1f 16783137

[20] Zare M, Javadi MA, Einollahi B, Karimian F, Rafie AR, Feizi S, et al. Changing Indications and Surgical Techniques for Corneal Transplantation between 2004 and 2009 at a Tertiary Referral Center. Middle East Afr J Ophthalmol. 2012;19:323–329. 10.4103/0974-9233.97941 22837628 PMC3401804

[21] Ang M, Wilkins MR, Mehta JS, Tan D. Descemet membrane endothelial keratoplasty. Br J Opthtalmology. 2016;100:15–21. 10.1136/bjophthalmol-2015-306837 25990654

[22] Ang M, Li L, Chua D, Wong C, Htoon HM, Mehta JS, et al. Descemet’s stripping automated endothelial keratoplasty with anterior chamber intraocular lenses: complications and 3-year outcomes. Br J Opthtalmology. 2014;98:1028–1032. 10.1136/bjophthalmol-2013-304622 24676725

[23] Ang M, Mehta JS, Lim F, Bose S, Htoon HM, Tan D. Endothelial Cell Loss and Graft Survival after Descemet’s Stripping Automated Endothelial Keratoplasty and Penetrating keratoplasty. Ophthalmology. 2012;119:2239–2244. 10.1016/j.ophtha.2012.06.012 22885122

[24] Patel S V. Graft survival and endothelial outcomes in the new era of endothelial keratoplasty. Exp Eye Res. 2012;95:40–47. 10.1016/j.exer.2011.05.013 21689649 PMC3902807

[25] Singh NP, Said DG, Dua HS. Lamellar keratoplasty techniques. Indian J Ophthalmol. 2018;66:1239–1250. 10.4103/ijo.IJO_95_18 30127133 PMC6113816

[26] Tourtas T, Laaser K, Bachmann BO, Cursiefen C, Kruse FE. Descemet Membrane Endothelial Keratoplasty Versus Descemet Stripping Automated Endothelial Keratoplasty. Am J Ophthalmol. 2012;153:1082–1090. 10.1016/j.ajo.2011.12.012 22397955

[27] Guerra FP, Anshu A, Price MO, Price FW. Endothelial Keratoplasty: Fellow Eyes Comparison of Descemet Stripping Automated Endothelial Keratoplasty and Descemet Membrane Endothelial Keratoplasty. Cornea. 2011;30:1382–1386. 10.1097/ICO.0b013e31821ddd25 21993468

[28] Woodward MA, Titus M, Mavin K, Shtein RM. Corneal Donor Tissue Preparation for Endothelial Keratoplasty. J Vis Exp. 2012;64:e3847. 10.3791/3847 22733178 PMC3671837

[29] Kitzmann AS, Goins KM, Reed C, Padnick-Silver L, Macsai MS, Sutphin JE. Eye Bank Survey of Surgeons Using Precut Donor Tissue for Descemet Stripping Automated Endothelial Keratoplasty. Cornea. 2008;27:634–639. 10.1097/QAI.0b013e31815e4011 18580252

[30] Ragunathan S, Ivarsen A, Nielsen K, Hjortdal J. Comparison of organ cultured precut corneas versus surgeon-cut corneas for Descemet’s stripping automated endothelial keratoplasty. Cell Tissue Bank. 2014;15:573–578. 10.1007/s10561-014-9429-x 24526412

[31] Terry MA. Endothelial keratoplasty: a comparison of complication rates and endothelial survival between precut tissue and surgeon-cut tissue by a single dsaek surgeon. Trans Am Ophthalmol Soc. 2009;107:184–193. 20126494 PMC2814585

[32] Regnier M, Auxenfans C, Maucort-boulch D, Marty A, Damour O, Burillon C, et al. Eye bank prepared versus surgeon cut endothelial graft tissue for Descemet membrane endothelial keratoplasty. Med. 2017;96:e6885. 10.1097/MD.0000000000006885 28489792 PMC5428626

[33] Mathes KJ, Tran KD, Mayko ZM, Stoeger CG, Straiko MD, Terry MA. Reports of Post-keratoplasty Infections for Eye Bank-prepared and Non–Eye Bank-prepared Corneas: 12 Years of Data From a Single Eye Bank. Cornea. 2019;38:263–267. 10.1097/ICO.0000000000001839 30601289

[34] Rauen MP, Goins KM, Sutphin JE, Kitzmann AS, Schmidt GA, Wagoner MD. Impact of eye bank lamellar tissue cutting for endothelial keratoplasty on bacterial and fungal corneoscleral donor rim cultures after corneal transplantation. Cornea. 2012;31:376–379. 10.1097/ICO.0b013e31823cbee3 22410614

[35] Ple-plakon PA, Shtein RM. Trends in corneal transplantation: indications and techniques. Curr Opin Ophthalmol. 2014;25:300–305. 10.1097/ICU.0000000000000080 24865170

[36] Montesel A, Alió del Barrio JL, Yébana Rubio P, Alió JL. Corneal graft surgery: A monocentric long-term analysis. Eur J Ophthalmol. 2020. 10.1177/1120672120947592 32757624

[37] Gómez-Benlloch A, Montesel A, Pareja-Aricò L, Mingo-Botín D, Michael R, Barraquer RI, et al. Causes of corneal transplant failure: a multicentric study. Acta Ophthalmol. 2021;1–7. 10.1111/aos.14708 33421330

[38] Melles GRJ, Lander F, van Dooren BTH, Pels E, Beekhuis WH. Preliminary Clinical Results of Posterior Lamellar Keratoplasty through a Sclerocorneal Pocket Incision. Ophthalmology. 2000;107:1850–1856. 10.1016/s0161-6420(00)00253-0 11013184

[39] Kymionis GD, Mikropoulos DG, Portaliou DM, Boboridis KG, Voudouragkaki IC, Dragoumis ND, et al. New Perspectives on Lamellar Keratoplasty. Adv Ther. 2014;31:494–511. 10.1007/s12325-014-0121-0 24846543

[40] Maier P, Reinhard T, Cursiefen C. Descemet Stripping Endothelial Keratoplasty—Rapid Recovery of Visual Acuity. Dtsch Arztebl Int. 2013;110:365–371. 10.3238/arztebl.2013.0365 23795211 PMC3679620

